# Discovery of Small-Molecule Modulators of the Human Y_4_ Receptor

**DOI:** 10.1371/journal.pone.0157146

**Published:** 2016-06-13

**Authors:** Gregory Sliwoski, Mario Schubert, Jan Stichel, David Weaver, Annette G. Beck-Sickinger, Jens Meiler

**Affiliations:** 1 Faculty of Biosciences, Pharmacy and Psychology, Institute of Biochemistry, Leipzig University, Leipzig, Germany; 2 Center for Structural Biology, Department of Chemistry, Vanderbilt University, Nashville, Tennessee, United States of America; 3 Department of Pharmacology, Vanderbilt University, Nashville, Tennessee, United States of America; University of North Dakota, UNITED STATES

## Abstract

The human neuropeptide Y_4_ receptor (Y_4_R) and its native ligand, pancreatic polypeptide, are critically involved in the regulation of human metabolism by signaling satiety and regulating food intake, as well as increasing energy expenditure. Thus, this receptor represents a putative target for treatment of obesity. With respect to new approaches to treat complex metabolic disorders, especially in multi-receptor systems, small molecule allosteric modulators have been in the focus of research in the last years. However, no positive allosteric modulators or agonists of the Y_4_R have been described so far. In this study, small molecule compounds derived from the Niclosamide scaffold were identified by high-throughput screening to increase Y_4_R activity. Compounds were characterized for their potency and their effects at the human Y_4_R and as well as their selectivity towards Y_1_R, Y_2_R and Y_5_R. These compounds provide a structure-activity relationship profile around this common scaffold and lay the groundwork for hit-to-lead optimization and characterization of positive allosteric modulators of the Y_4_R.

## Introduction

Obesity, a major risk factor for diabetes, heart disease, cancer, and mortality is a rising medical concern with doubled worldwide prevalence since 1980, reaching an estimated medical cost of $147 billion in 2008 [1, 2]. Dietary changes and nutritional counseling can be effective treatment options but results are inconsistent, suffering from poor long-term patient adherence often leading to weight regain [3]. So far, only invasive treatments such as bariatric surgery show long term success rates, but are limited to patients where the benefits outweigh the risks and costs [4]. Several studies suggest that hormonal changes following bariatric surgery contribute to its long term success [2, 5]. Their respective hormone receptors may represent promising therapeutic targets. For example, meal-stimulated glucagon-like peptide-1 (GLP-1) release is thought to participate in the long-term success of bariatric procedures. GLP-1 receptor agonists have been shown to produce weight loss and glucose homeostasis for subjects with type II diabetes. However, the weight loss seen with GLP-1 agonists alone is modest [6].

Two members of the pancreatic polypeptide family including peptide tyrosine tyrosine (PYY) and pancreatic polypeptide (PP) act as satiety factors to inhibit food intake, and modify metabolic homeostasis [7]. Along with the third member of this class of hormones, neuropeptide Y (NPY), the peptides regulate energy metabolism through four different Y receptor subtypes in humans: Y_1_R, Y_2_R, Y_4_R and Y_5_R. All receptor subtypes are involved in the regulation of energy metabolism and are putative targets for the treatment of obesity. Furthermore, in this multi ligand/multi receptor system, the receptor subtypes display different preferences for NPY, PYY and PP. Y_1_R, Y_2_R and Y_5_R bind NPY and PYY with high affinity. In contrast, PP is strongly preferred by the Y_4_R and binds to this receptor subtype with a high affinity and with lower affinity to the Y_5_R [8].

While PP and PYY both present promising routes for the treatment of obesity, PP may be preferred as it inhibits feeding in mice more than PYY and PYY-3-36 [9]. Pancreatic polypeptide has also been shown to inhibit food intake in humans [10]. Further, in contrast to PP, medically relevant doses of PYY induce nausea in humans [10, 11].

PP is released under vagal cholinergic control from F-cells of pancreatic islets in response and proportion to food ingestion [12]. The hormone is furthermore expressed in some endocrine cells of the intestines [13]. Primarily through Y_4_R, PP promotes appetite suppression, inhibition of gastric emptying, and increased energy expenditure [14]. The human Y_4_R is a 375 amino acid class A G-protein coupled receptor (GPCR) primarily expressed in the gastrointestinal tract, where it inhibits peristalsis and excretion [15]. Other peripheral organs that express Y_4_R include the heart, skeletal muscle, and thyroid gland. In the central nervous system, Y_4_R is expressed in the hypothalamus, where it relays anorexigenic signals [16] and inhibits neurotransmitter release [17]. The Y_4_R is a putative target for the treatment of obesity based on its strong anorexigenic potential and studies involving the overexpression or endogenous application of PP [16, 18–20].

Our efforts focus on the identification of small-molecule positive allosteric modulators (PAMs) of Y_4_R. Allosteric ligands represent promising options for treatment of metabolic and neurological diseases [21]. Allosteric ligands show a range of pharmacological activities including PAMs (agonism, potentiation or both), negative allosteric modulation (NAM), and inverse agonism. These ligands have the potential to differentially regulate several pathways on the same GPCR and induce a biased signaling [22]. Additionally, PAMs with little or no intrinsic activity may be safer therapeutics because their dependence on the presence of the endogenous agonist may help to prevent toxicity and other negative side effects [23]. This approach preserves the physiological signaling patterns, which may be critical in complex systems and is not feasible using orthosteric agonists [24]. Allosteric binding sites may be less conserved between receptor subtypes than the orthosteric binding site because they lack the evolutionary pressure to conserve affinity for the orthosteric ligand. Therefore it is often possible to design allosteric modulators with high selectivity [24, 25].

Since no small-molecule PAM or agonist has been identified for Y_4_R, we used high-throughput screening (HTS) [26] to identify compounds that modulate the Y_4_R. Initial hit compounds were validated as PAMs in a complementary set of assays and subtype-selectivity was investigated for all human NPY receptors.

## Materials and Methods

### Cell Culture

COS-7 cells stably expressing hY_1/2/4/5_R_eYFP fusion protein and the Δ6Gα_qi4myr_ chimeric Gα-protein were prepared as previously described [27]. The hY_1/2/4/5_R_eYFP cDNA was subcloned into MCS1 of a pVitro2-MCS vector carrying a hygromycin resistance gene; the Δ6Gα_qi4myr_ cDNA was subcloned into MCS1 of a pVitro2-MCS vector carrying a G418 resistance gene (Invivogen, San Diego, CA).

COS-7 (African Green Monkey kidney) cells stably expressing the hY_1/2/4/5_R_eYFP fusion protein and the Δ6Gα_qi_4myr chimeric Gα-protein were cultured at 37°C in high glucose Dulbecco’s Modified Eagle Medium (DMEM, Life Technologies, Carlsbad, CA) with glutamine and sodium pyruvate (Life Technologies/Lonza) supplemented with 10% (w/v) FBS (Invitrogen, Carlsbad, CA), 1.5 mg/mL G418-sulfate (Amresco, Solon, OH) and 133 μg/ml hygromycin (Invivogen).

### HTS Calcium Flux Assay

The purities of compounds in the Vanderbilt HTS facility are more than 95% as confirmed by the supplier. A total of 35,288 compounds were tested for their ability to modulate the Y_4_R activation in conjunction with the Vanderbilt HTS facility. In a pilot HTS experiment, 2000 compounds (spectrum collection, MicroSource Discovery Systems, Inc., Gaylordsville, CT) were screened for modulation of the Y_4_R. This collection is designed to enrich the general hit rate by including drugs with known biological profiles (60%), naturally occurring products with no biological profile (25%), and non-drug compounds with biological profiles (15%). In a following HTS, 33,288 compounds were tested for Y_4_R modulatory effects. Thirty-two thousand of these compounds were randomly selected from the Vanderbilt compound library and 1288 were selected based on their similarity to Niclosamide, a PAM discovered in the pilot HTS.

Cells were plated in TC-treated 384-well plates (black, clear bottom, Greiner, Monroe, NC) in 20 μL cell culture medium using a Multidrop Combi (Thermo Fisher, Waltham, MA) microplate dispenser (ThermoScientific, Thermo Fisher) at a density of 16,000 cells/well. The cells were incubated for 24 hours at 37°C in the presence of 5% CO_2_. Following incubation, the medium was replaced with 20 μL/well fluorescent dye solution (1.0 μM Fluo-2 AM (TEFlabs, Austin, TX), .01% (v/v) Pluronic Acid F-127 in assay buffer) using an ELx405 cell washer (BioTek, Winooski, VT). Following a 90 minute incubation at room temperature, fluorescent dye solution was replaced with 20 μL/well assay buffer (HBSS, 20 mM HEPES, and 1.25 mM Probenecid (Sigma-Aldrich, St. Louis, MO)) using the ELx405 and cell plates were loaded into a Functional Drug Screening System (FDSS, Hamamatsu, Japan). Once loaded into the FDSS, cell plates were imaged at 1Hz (excitation 470 ± 20 nm, emission 540 ± 30 nm using a 3-addition protocol designed to detect agonists, potentiators, and inhibitors: 1) after collecting 4 seconds of baseline, 20 μl/well of 20 μM test compounds in assay buffer + 0.1% (w/v) fatty-acid-free bovine serum albumin (Sigma-Aldrich, modified assay buffer) were added 2) following a 150 second delay, 10 μl/well of concentration of 5-fold over the PP EC_20_ (55 ± 27 pM) 3) after 330 seconds, a 13 μl/well addition 5-fold over the PP EC_80_ (836 ± 33 pM) in modified assay buffer was performed. On each screening day, PP EC_20_ and EC_80_ plates were adjusted after a test PP CRC at the beginning of each day to account for minor day to day variations in experimental conditions.

### Substructure Search

Substructure searches were performed against the Vanderbilt Institute for Chemical Biology (VICB) library using the ChemCart application (DeltaSoft Inc., Hillsborough, NJ) Tanimoto coefficient similarity search. Higher Tanimoto coefficients indicate more similarity based on the shared presence of chemical subgroups. To increase our chemical search space, we altered the amide linker of Niclosamide and repeated the substructure search against the VICB library. Linker alterations included replacing the amide linker with urea, thiourea, δ-lactam, and an extension of the linker by one or two methylene groups. Tanimoto coefficient cut-offs were adjusted to between 0.45 and 0.63 for each substructure search to ensure that approximately 200 to 300 compounds were identified for each scaffold (S1 Table). Reference structures with alternate backbone constitutions were generated using ChemBioDraw Ultra (PerkinElmer Inc., Waltham, MA). Final search results were concatenated and duplicate search hits were removed. The final collection of 1288 compounds were distributed over five plates and tested using the triple-add screen protocol.

### IP_3_ Assay

Cells were seeded into 48-well plates and incubated for 24 hours at 37°C / 5% CO_2_. Afterwards, cells were labeled for at least 16 hours in DMEM+10% (w/v) FBS containing 2 μCi/ml *myo*-[2- ^3^H(N)]-inositol (PerkinElmer) at 37°C / 5% CO_2_. Labeling solution was aspirated and cells were washed with 20 μL/well DMEM + 10 mM LiCl (Sigma-Aldrich; DMEM/LiCl) and stimulated with the peptide solutions and compounds. Specifically, 50 μl/well DMEM/LiCl were added after washing, followed by addition of 50 μl/well test compound in DMEM/LiCl (3-fold over the final concentration) and addition of 50 μl/well peptide solution (3-fold over the final concentration) in DMEM/LiCl. Stimulation was performed for 2 hours at 37°C / 5% CO_2_. Cell lysis, subsequent sample preparation and radiometric detection was performed as described previously [27].

### Data analysis

Data analysis was performed with GraphPad Prism 5.03 software (GraphPad Software, San Diego, CA). Calculation of EC_50_ and pEC_50_ was performed using standard non-linear regression (log(agonist) vs. response, three parameters). All data were normalized to the corresponding control curve in the absence of the modulator. Final concentration response curves were created by calculating the mean and SEM of all individual experiments for each data point. Statistical evaluation was performed using two-way ANOVA and Bonferroni post test with * P ≤ 0.05, ** P ≤ 0.01, *** P ≤ 0.001.

## Results

### Identification of Y_4_R PAMs

**Fig 1:**
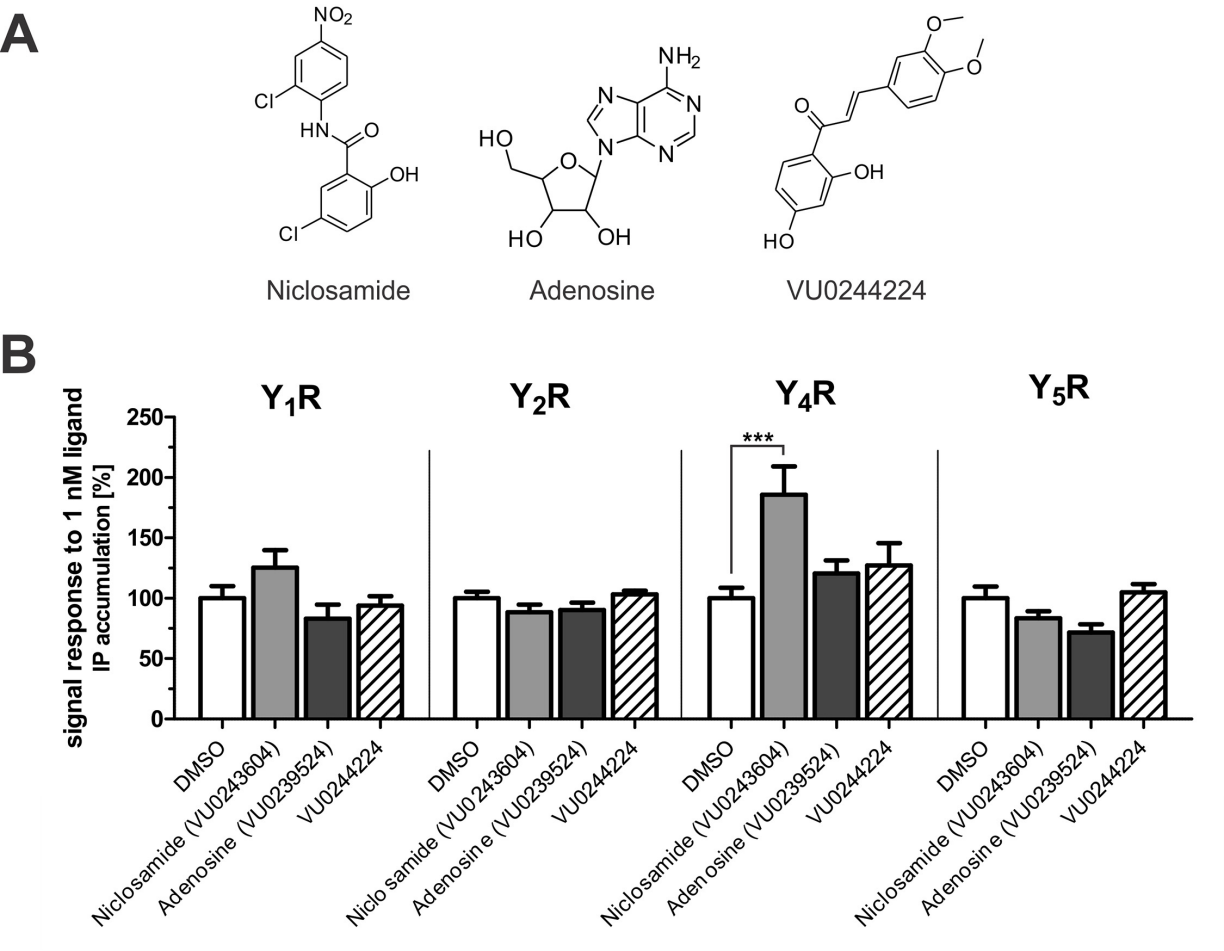
Validation of Y_4_R PAM activity and subtype selectivity of initial Ca^2+^-flux-based screen hit compounds in an inositol phosphate accumulation assay. (A) Compound structures. (B) Effect of 10 μM compound on submaximal YR activation by 1 nM ligand, which represents EC_20_-EC_60_ (Y_1,2,5_R: NPY; Y_4_R: PP). Data represent the mean ± SEM of two independent experiments each performed in quadruplicate (*** p ≤ .001 Bonferroni).

Until now, no small molecule agonists of Y receptors have been described. Based on this lack of any structure activity data, identification of Y_4_R PAMs was initiated with a Ca^2+^-flux-based HTS approach. An initial pilot screen was performed with the ‘Spectrum collection’, a small scale library with 2000 compounds comprising synthetic small molecules as well as purified natural products covering a range of known biologically active properties. This pilot screen yielded 65 putative PAMs. All initial hits were retested for off-target effects in wildtype COS-7 cells and the PAM effect was validated via concentration-dependent potentiation of a submaximal PP response. After eliminating structures that show off-target effects, seven compounds with potencies in the micromolar range were identified (S1 Fig) for further investigation. Validation of the Y_4_R PAM activity was performed with a well-established assay system for Y receptor activation studies, based on cellular accumulation of inositol phosphate [28, 29]. Furthermore, the compound activity at Y_1_R, Y_2_R and Y_5_R was examined at this stage of Y_4_R PAM identification. Therefore, Y receptor activation with an agonist ligand concentration causing a submaximal response (1 nM, Fig 1) was monitored in presence and absence of test compound. This experimental setup allows the detection of potential compound-induced shifts of the concentration-response curve and parallel testing of all compounds on all Y receptor subtypes. Cells stably expressing the chimeric G-protein Δ6G_αqi4myr_ and one of four different human NPY receptor subtypes (Y_1_R, Y_2_R, Y_4_R, or Y_5_R) were treated with test compound at a final concentration of 10 μM followed immediately by stimulation with 1 nM endogenous peptide agonist (Y_4_R: PP, Y_1,2,5_R: NPY).

**Fig 2:**
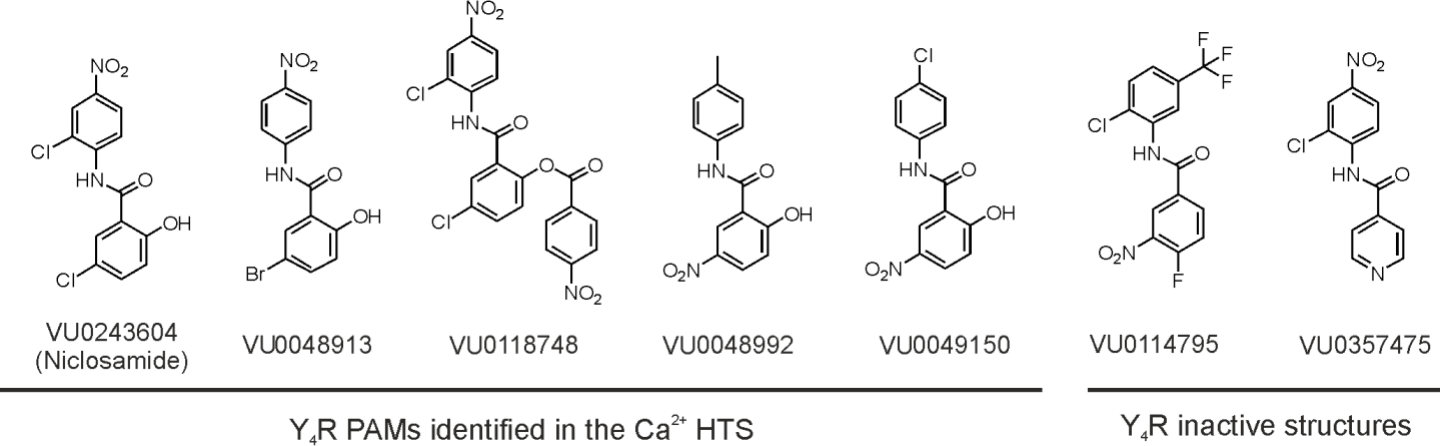
Structures of Y_4_R PAMs identified by HTS and inactive control compounds chosen for further characterization of Y_4_R PAM activity and YR subtype selectivity.

The effect of the compounds on inositol phosphate accumulation was investigated in relation to the control in presence of DMSO (DMSO set to 100%, complete data of all 7 compounds see S1 Fig). However, only three of the seven HTS PAM hits validated their Y_4_R PAM effect also in the inositol phosphate accumulation assay (Fig 1). Of all tested compounds, Niclosamide was the strongest Y_4_R PAM, significantly increasing IP accumulation following stimulation of Y_4_R with 1 nM PP (185.7 ± 23.5% (SEM) versus 100.00 ± 13.9%, p<0.05, n = 2; see Fig 1). Furthermore, Niclosamide had minor effects on Y_1_R (125.4 ± 14.6% versus 100.0 ± 28.3%, p>0.05, n = 2), Y_2_R (88.5 ± 6.2% versus 100.0 ± 14.9%, p>0.05, n = 2), and Y_5_R (83.4 ± 5.9% versus 100.0 ± 27.3% p>0.05, n = 2) when stimulated with 1 nM NPY. In addition to Niclosamide, adenosine and VU0244224 induced slight increases in the Y_4_R signal response in the IP_3_ assay (120 ± 11% and 127 ± 19%, respectively). Whereas VU0244224 had no effects on other Y receptor subtypes, adenosine appeared to slightly decrease signal transduction at Y_1_R and Y_5_R. These results show Niclosamide to be the most effective Y_4_R PAM hit compound with validated activity in a complementary assay and it alone was selected for further investigation (S1 Fig). Due to the lack of any viable hits with the exception of Niclosamide, a second screen was performed testing a total of 33,288 compounds. Of these 33,288 compounds, 32,000 were randomly selected from the VICB compound library. Since Niclosamide showed the strongest effect as a Y_4_R PAM, the collection of compounds for the second screening was enriched with 1,288 compounds structurally similar to Niclosamide based on Tanimoto coefficients (S1 Table). All potential Y_4_R PAMs were retested for nonspecific effects in wildtype COS-7 cells. Hit compounds that did not show any activity in wildtype COS-7 cells were tested on the Y_4_R in a concentration dependent manner for their effect on a PP EC_20_ and EC_80_. Validated hits were then tested for their effects on histamine and bradykinin receptor-evoked changes in intracellular Ca^2+^ in order to further exclude off-target effects that might, for instance, result from changes in common effectors downstream of the GPCR. In the second HTS, four compounds structurally similar to Niclosamide were identified as Y_4_R PAMs. Of these similar compounds, stability of VU0118748 was examined to ensure that activity was not due to hydrolysis yielding Niclosamide. HPLC analysis at different incubation times suggest that this compound is stable (S3 Fig). Along with two structurally related but inactive compounds of the VU compound library (VU0114795 and VU0357475), selected to supplement structure-activity relationship studies with YR selectivity, these compounds were further investigated for Y_4_R PAM activity and YR subtype selectivity (Fig 2).

### Validation and selectivity of Y_4_R PAMs

**Fig 3:**
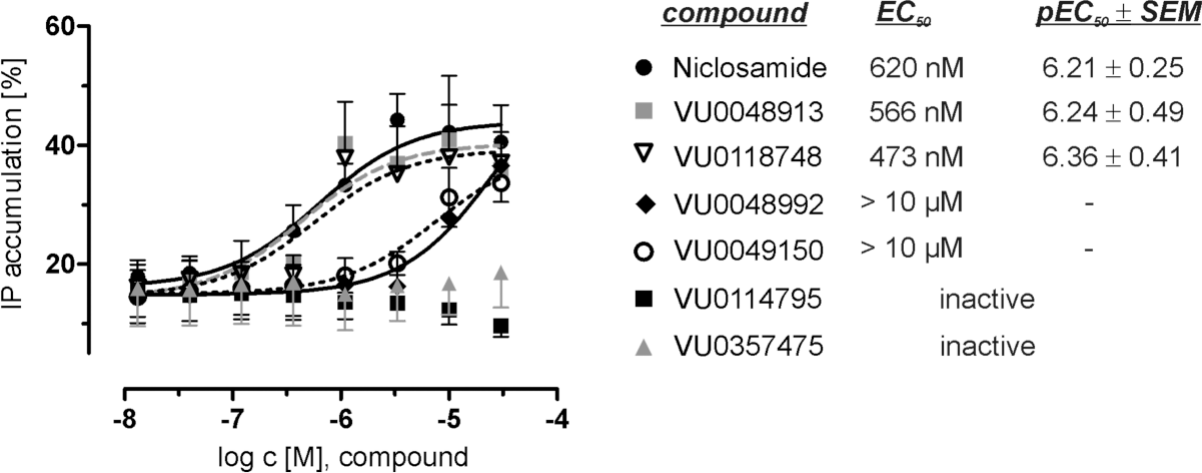
Y_4_R PAM activity of Niclosamide-like compounds. Potency of the Y_4_R PAMs was investigated with an inositol phosphate accumulation assay through potentiation of a PP EC_20_ response. Data have been normalized to the maximum IP accumulation caused by the Y_4_R native ligand PP. Data represent the mean ± SEM of three independent experiments performed in duplicate.

After identification of Niclosamide-like compounds in the HTS, the Y_4_R PAM activity was again validated using an IP_3_ accumulation assay system. Compounds were investigated for potentiation of a PP EC_20_ in a concentration-dependent manner to determine their potency on the Y_4_R (Fig 3).

Niclosamide, VU0048913, VU0118748, VU0048992 and VU0049150, identified in the Ca^2+^ flux HTS, potentiated the PP signal response in the IP_3_ accumulation assay. In presence of 30 μM of the active compounds, the PP response increased to approximately 40% (Fig 3) compared to the PP-evoked signal in the absence of the compounds. However, modifications on the Niclosamide scaffold affected the potency of the compounds. Niclosamide, VU0048913, and VU0118748 potentiated the PP EC_20_ with comparable EC_50_ values of 620 nM, 566 nM and 473 nM, respectively. In contrast, structural modifications in compound VU0048992 lead to a dramatic loss of Y_4_R potency (EC_50_ >10 μM) or completely inactive structures in case of VU0114795 and VU0357475.

**Fig 4:**
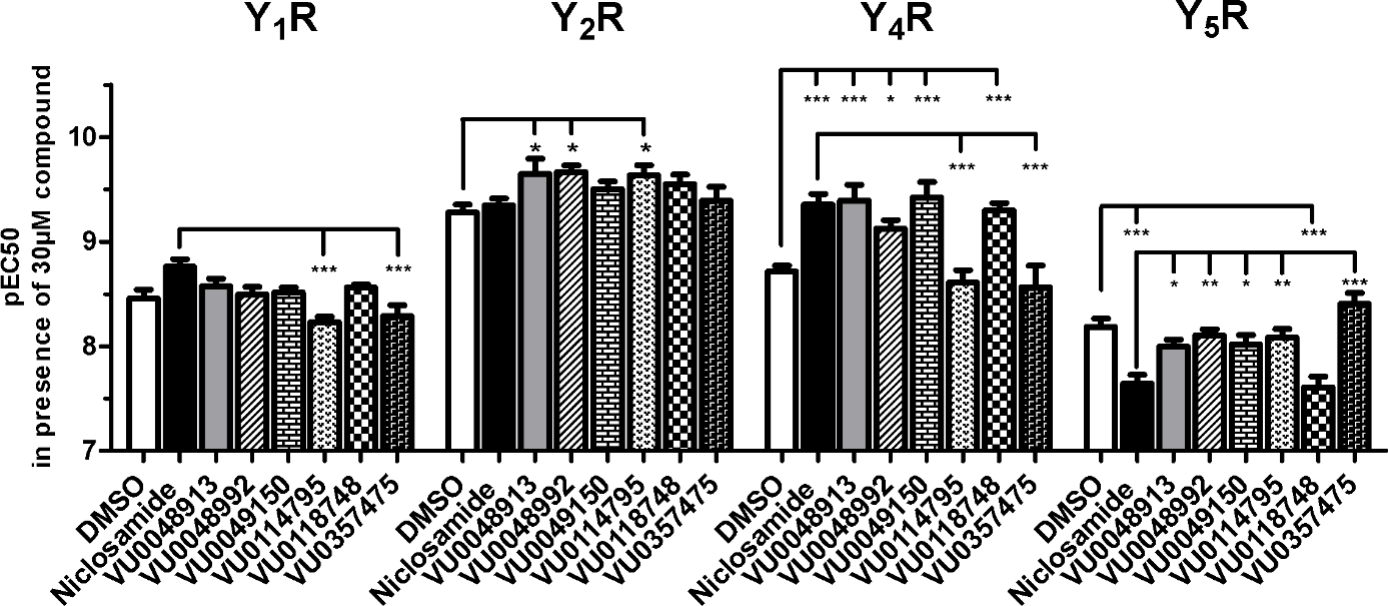
YR subtype selectivity of Y_4_R PAMs. Effect of 30 μM compound on the pEC_50_ of Y-receptor agonists in COS-7 cells stable expressing a Y receptor subtype and the chimeric G-protein G_α6qi4myr_. Receptors were stimulated with their native ligands (Y_1_R, Y_2_R, Y_5_R: NPY; Y_4_R: PP). For Y-axis values, positive modulation represents an increase in the apparent potency (pEC_50_) of the native agonist and negative modulation represents a decrease in the apparent potency of the native agonist. Data represent the mean ± SEM of at least two independent experiments (for full concentration-response curves see S2 Fig) (*p < .05, ***p < .001 Bonferroni).

To investigate the Y receptor subtype selectivity, we tested the effect of the Niclosamide-like structures (Fig 2) at all four human Y receptors with their native ligands (PP for Y_4_R and NPY for Y_1_R, Y_2_R, and Y_5_R). Full concentration-response relationships were determined for each ligand-receptor pair in the presence of DMSO vs. 30 μM compound (S2 Fig), the concentration at which all compounds had a comparable effect on the Y_4_R (Fig 3). This high compound concentration was used for response curves in S2 Fig due to failure of some compounds to reach maximal response in concentrations used in Fig 3. None of the tested compounds had an effect on the basal level or maximum level of the signal response (S2 Fig). Thus, the influence of the compounds on the agonists EC_50_ (pEC_50_ ± SEM) of the signal response was used as an indicator for selectivity (Fig 4).

Niclosamide, VU0048913, VU0048992, VU0049150, and VU0118748 increase the potency of PP at the Y_4_R (Fig 4, S2 Fig) consistent with Y_4_R PAM activity observed (Fig 3). Testing of other Y receptor subtypes revealed that the compounds are not fully selective for the Y_4_R subtype. In addition to the Y_4_R activity, Niclosamide had a small PAM effect on the Y_1_R. However, the structural analogs VU0048913, VU0048992, VU0049150, and VU0118748 had no effect on the Y_1_R signal. In contrast to the PAM effects observed on Y_4_R, Niclosamide and VU0118748 show a negative allosteric effect on Y_5_R. All other tested compounds were inactive on the Y_5_R. Whereas Niclosamide had no effect on the Y_2_R, compounds VU0048913, VU0048992, and VU0114795 showed a slight PAM effect on the Y_2_R. Overall, the effects of the Y_4_R PAM compounds on Y_1_R, Y_2_R, and Y_5_R were lower than the PAM effect at the Y_4_R.

### Niclosamide structure-activity relationships

**Fig 5:**
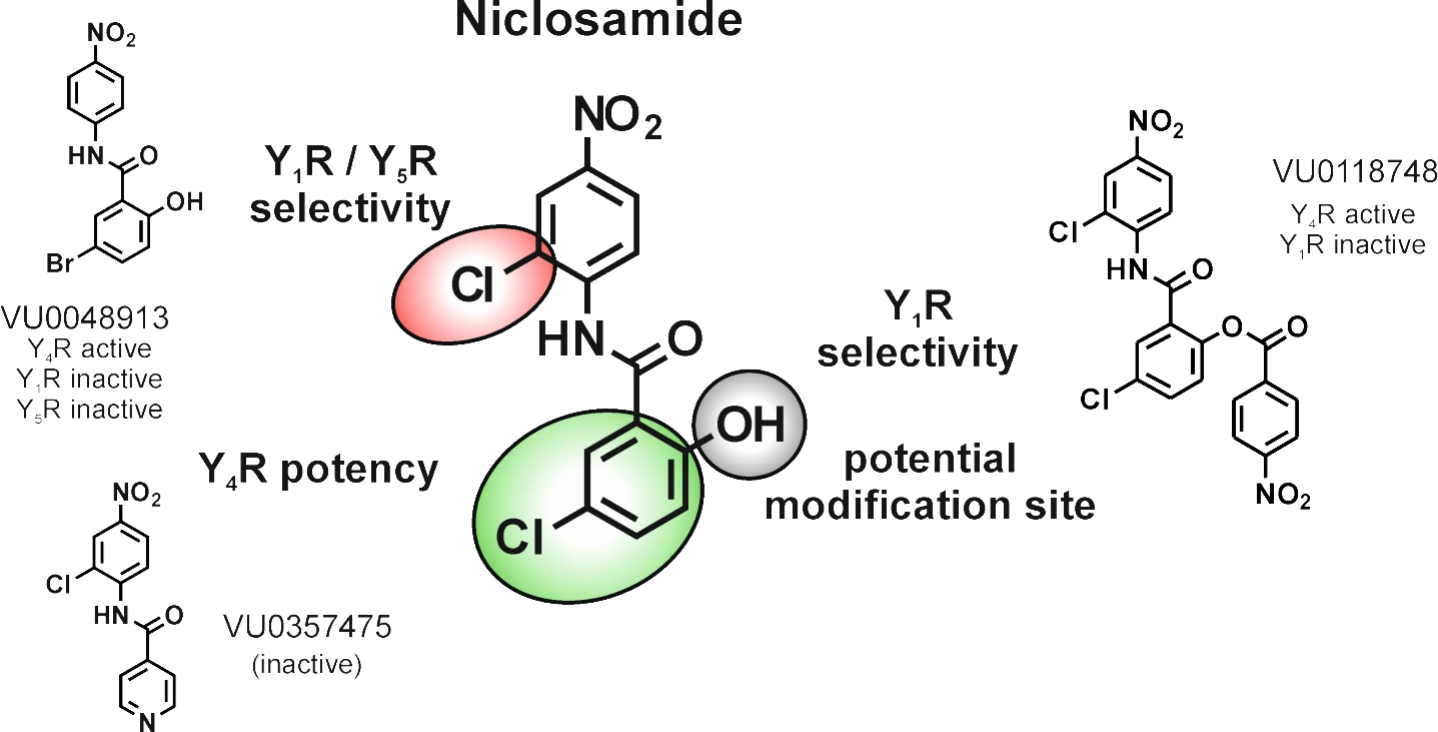
Distinct positions of the Niclosamide scaffold were shown to be relevant for Y_4_R PAM activity and YR selectivity. Substitutions in the benzoyl ring are important for Y_4_R potency (green), and offer a potential modification site (grey). Modifications in the aniline ring engender selectivity towards Y_1_R / Y_5_R subtype (red).

Potentiation of PP EC_20_ experiments showed that Niclosamide, VU0048913, and VU0118748 have nearly identical potencies at the Y_4_R. Accordingly, the loss of the Cl atom (VU0048913) on the aniline ring structure, as well as modification of the OH group in the benzoyl ring (VU0118748) do not affect Y_4_R PAM activity. In contrast, a major change of the substituents meta or para to the hydroxyl function on the benzoyl ring led to a complete loss of Y_4_R PAM activity (VU0114795 and VU0357475) or drastically reduced Y_4_R potency (VU0048992 and VU0049150). As shown by the active compound VU0048913, the Cl on the benzoyl ring structure can be substituted by Br, which underlines the importance of the electron-rich character in this position for the potency to the Y_4_R (Figs 3 and 5).

Furthermore, investigation of PAM activity on Y_1_R, Y_2_R and Y_5_R suggested potential modification sites to control YR selectivity. Removal of the Cl substitution on the aniline ring in VU0048913 reduced Y_1_R effects and Y_5_R antagonism. As shown by VU0118748, selectivity towards Y_1_R can also be achieved by the introduction of the nitrobenzoic-acid on the OH position in the benzoyl ring. However, this modification had no effect on the Y_5_R NAM activity and Y_4_R PAM activity, suggesting this position as a potential site to control subtype selectivity.

## Discussion

### Therapeutic potential

The application of allosteric modulators presents a promising approach for the treatment of complex receptor-ligand systems that regulate sensitive physiological processes such as nervous signal transduction or metabolic regulation [21, 30]. The benefits of Y_4_R modulation on obesity and insulin resistance are becoming more important as the number of patients diagnosed with type 2 diabetes rises alongside risk factors such as the prevalence of obesity, physical inactivity, and poor diet [31]. In humans, low circulating PP levels were found in obese children and adults [32]. Hyperphagia in obese patients can be reduced by restoring basal and meal-stimulated PP levels through IV infusion [33]. Additionally, PP is hypothesized to sensitize the liver to insulin through upregulation of the insulin receptor β subunit [34, 35]. In patients with diabetes secondary to chronic pancreatitis, PP administration reduces insulin resistance and improves glucose metabolism [36]. Effects of PP administration and the complex interplay of obesity and diabetes suggest Y_4_R modulation may be beneficial for a wide range of metabolic disorders. This is already being seen in preclinical studies of TM-30339, a PP based Y_4_R-selective peptide agonist and in phase I and II trials of Obineptide, an Y_2/4_R dual peptide agonist [37]. With respect to these diverse effects of the PP dependent Y_4_R activation, the availability of small molecule ligands and modulators offer a new possibility to get further insights into the pharmacology of this GPCR.

In this study, we present the first small molecule Y_4_R PAM. Niclosamide was identified as a Y_4_R PAM in the primary screen of the Spectrum Collection using the Ca^2+^ flux assay and its activity was validated in the alternative IP accumulation assay. The second HTS experiment identified four additional Y_4_R PAMs that are structurally similar to Niclosamide, confirming the importance of this scaffold for Y_4_R potentiation. YR subtype selectivity was characterized for four Niclosamide-like Y_4_R PAMs along with two Y_4_R inactive Niclosamide-derived structures.

### Y_4_R potency and selectivity

Investigation of the potency of the compounds on the Y_4_R showed that three compounds, Niclosamide, VU0048913, and VU0118748, had comparable EC_50_ values of around 500 nM. All other compounds lacking the halogen substitution on the benzoyl ring were either completely inactive at the Y_4_R or had an at least 10-fold lower EC_50_ for potentiation of a PP EC_20_ (Fig 3). These investigations highlight the role of an electron-rich substituent at this position for Y_4_R potency. In contrast, the bulky nitro-benzoyl substitution in VU0118748 had no influence on Y_4_R potency and Y_4_R PAM activity. However, this modification lowers the effect at Y_1_R. This offers a role for this position as a potential modification site for improving Y_4_R selectivity while maintaining the Y_4_R PAM activity.

Selectivity studies of Niclosamide and analogs on all four Y receptor subtypes revealed that the compounds are not fully selective for the Y_4_R. NPY was used as the ligand for selectivity studies on Y_1_R, Y_2_R and Y_5_R, as it activates all three receptors with high potencies. However, allosteric modulators are known for the potential of probe dependence effects for different ligands of one GPCR and thus effects for alternative NPY ligands PYY or PP on these Y receptor subtypes cannot be excluded at this stage of the investigations. Niclosamide and VU0118748, both Y_4_R PAMs, showed antagonistic effects on the Y_5_R. The Y_4_R and Y_5_R fulfill different actions in the regulation of appetite and food intake. While the Y_4_R has an anorexigenic effect by inducing satiety in response to the activation of the native ligand PP, the Y_1_R and Y_5_R are characterized to have an orexigenic effect [38]. A simultaneous Y_5_R antagonism and Y_4_R PAM activity thus could contribute to an anti-obesity effect of Niclosamide or VU0118748. However, the different analogs of the compound Niclosamide scaffold suggest the possibility of developing Y_4_R PAMs with a higher degree of specificity relative to other Y receptor subtypes (Fig 5). It is not uncommon for compounds to produce a variety of effects at a common binding site. For example, recently a known metabotropic glutamate receptor 4 (mGluR4) PAM/mGluR1 NAM chemotype was converted into a selective mGluR1 PAMs by virtue of a double “molecular switch” [39].

### Would an EC_50_ shift of 4 fold be sufficient to cause an *in vivo* effect?

Niclosamide and some related analogs induced a 4-fold increase in the potency of PP at Y_4_R. Allosteric modulators of other class A GPCRS, especially of neurotransmitter receptors, display a stronger allosteric effects with EC_50_ shifts >10 fold, shown for muscarinic receptor 4 (mAChR4) [40]. However, allosteric modulation of the CaSR by cinacalcet shows that even smaller *in vitro* effects can be effective *in vivo* and that clinical efficacy is dependent on the receptor, tissue and the metabolic state that is targeted [41]. This suggests that the comparatively small increase in PP potency caused by Niclosamide may be sufficient to elicit *in vivo* effects.

### Niclosamide improves diabetic symptoms in mice

Niclosamide is an FDA approved anthelmintic drug that treats parasitic worm infection through the uncoupling of mitochondria. Interestingly, this compound was recently studied as a potential therapeutic for treatment of type 2 diabetes due to its high tolerability and the benefits of lipid mitochondrial uncoupling for treating diabetes [42]. Tao et al. fed mice the ethanolamine salt form of Niclosamide and showed it to be efficacious at high nanomolar concentrations (measured with blood sample liquid chromatography–tandem mass spectrometry at various time points) in reducing plasma insulin decline in db/db mice, sensitizing the insulin response, and preventing and treating diabetic symptoms during high fat diet induced obesity in mice. The authors focus on mitochondrial uncoupling as the primary mechanism of action for Niclosamide in the treatment of diabetes symptoms. However, our identification of Niclosamide as a Y_4_R PAM suggests that the efficacy of Niclosamide on diabetic symptoms may instead result from its action on the YR signalling in addition to effects on mitochondrial function.

## Supporting Information

S1 Fig Validation Y_4_R PAM activity and YR subtype selectivity of initial Ca^2+^ HTS hit compounds in an IP_3_ assay. Structurally different small molecules (A) showed a positive effect on Y_4_R Ca^2+^ signal response in an HTS screening of the spectrum collection. Retesting in the IP_3_ assay as an alternative YR activation readout validated Niclosamide as a Y_4_R PAM (B) and offered other hits to have additional effects on other YR subtypes. Submaximal activation of Y receptors was observed for stimulation with 1 nM ligand (Y4R: PP, Y_1_,_2_,_5_R: NPY) in presence of 10 µM compound. Data represent the mean ± SEM of two independent experiments performed in quadruplicates (***p < .005 Bonferroni).
(TIF)

S2 Fig Selectivity of Niclosamide-like allosteric modulators among human Y receptors. Receptor activation was investigated with an inositol phosphate accumulation assay in COS-7 cells stably expressing a Y receptor subtype and chimeric G-protein ΔGα6qi4myr. Data represent the mean ± SEM of at least 2 independent experiments, each performed in triplicate.
(TIF)

S3 Fig HPLC analysis of VU0118748 stability in DMEM assay conditions. Niclosamide and VU0118748 showed different retention times of 46 min (63% ACN) and 58 min (71% ACN), respectively. VU0118748 in DMEM (without pre-incubation, black) shows a major signal with 98% integrated absorption at 58 min retention time, indicating the intact VU0118748. To test the stability of VU0118748 in the assay conditions, the compound was pre-incubated in DMEM for 2 hours at 37°C. HPLC analysis afterwards (green) shows a slightly increased fraction at 46 min retention time (5% of total absorption). Results show 95% of the compound is still intact.
(PNG)

S1 Table Substructure search: Niclosamide analogues and results. (PDF)
