# Supplementary figures and images for: Discovery of Small-Molecule Modulators of the Human Y4 Receptor
Source: PLoS One. 2016 Jun 13;11(6):e0157146. doi: 10.1371/journal.pone.0157146 (PMC4905667; doi:10.1371/journal.pone.0157146)

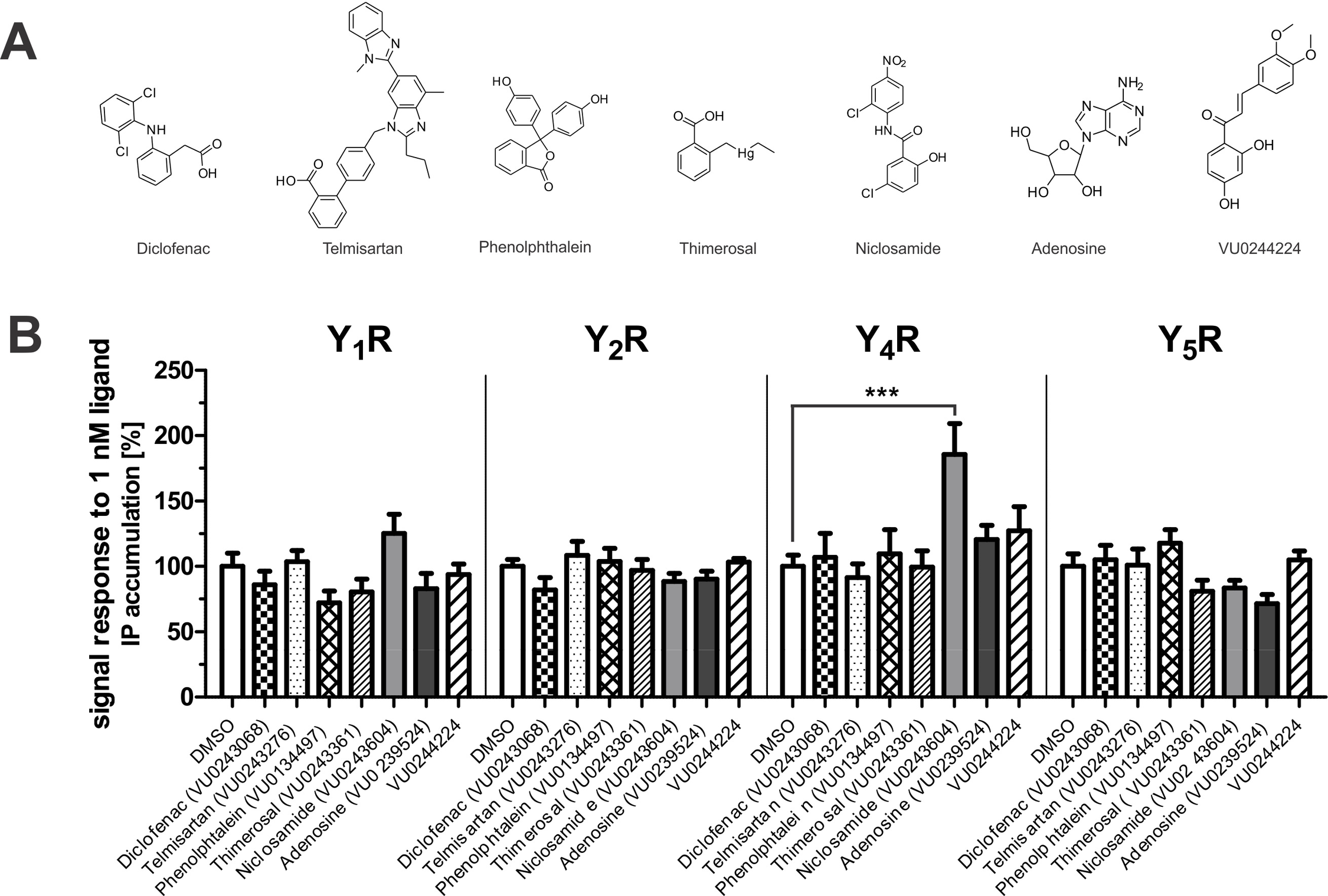

Supplement: S1 Fig — Structurally different small molecules (A) showed a positive effect on Y4R Ca2+ signal response in an HTS screening of the spectrum collection. Retesting in the IP3 assay as an alternative YR activation readout validated Niclosamide as a Y4R PAM (B) and offered other hits to have additional effects on other YR subtypes. Submaximal activation of Y receptors was observed for stimulation with 1 nM ligand (Y4R: PP, Y1,2,5R: NPY) in presence of 10 µM compound. Data represent the mean ± SEM of two independent experiments performed in quadruplicates (***p < .005 Bonferroni). (TIF) [file pone.0157146.s001.tif]

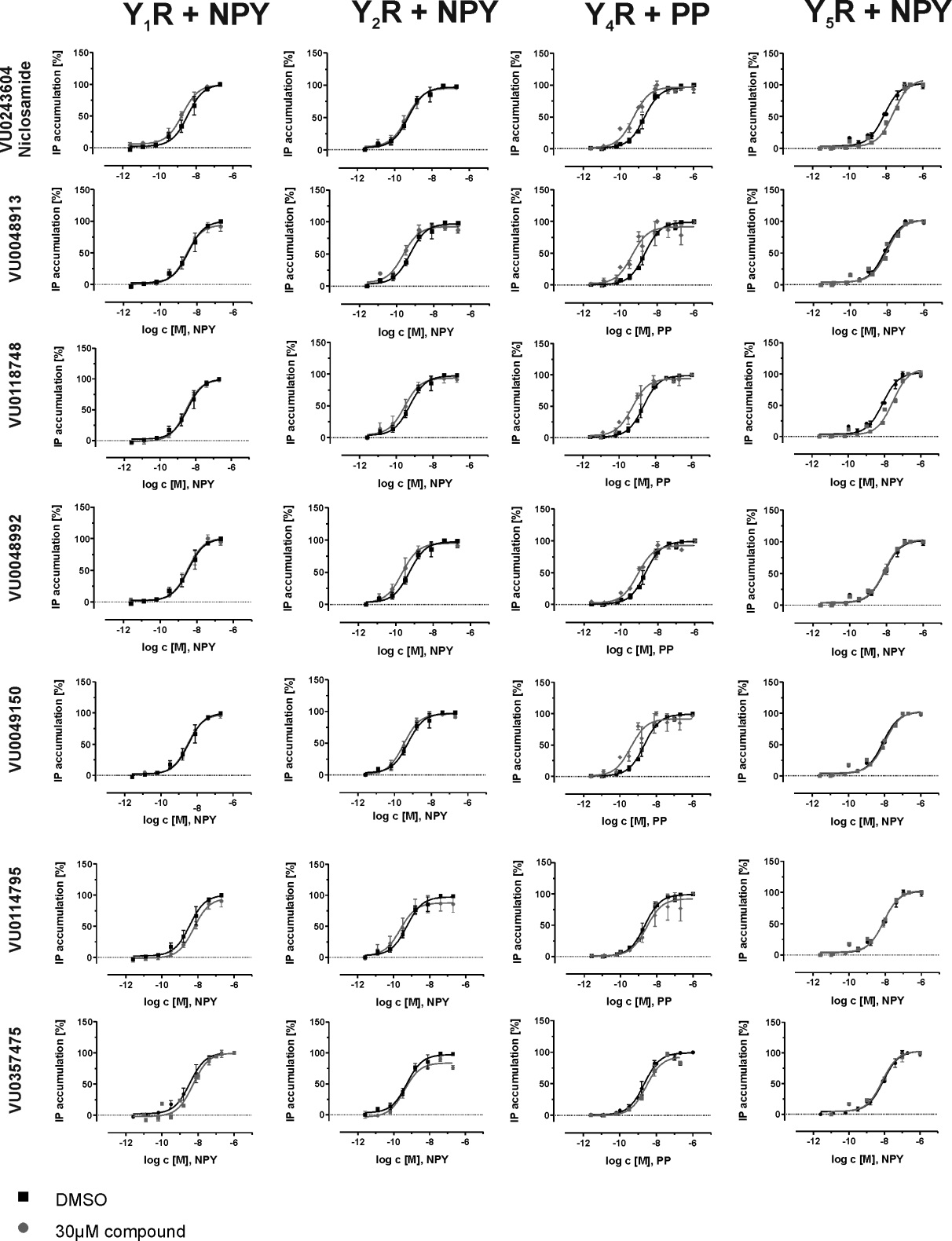

Supplement: S2 Fig — Receptor activation was investigated with an inositol phosphate accumulation assay in COS-7 cells stably expressing a Y receptor subtype and chimeric G-protein ΔGα6qi4myr. Data represent the mean ± SEM of at least 2 independent experiments, each performed in triplicate. (TIF) [file pone.0157146.s002.tif]

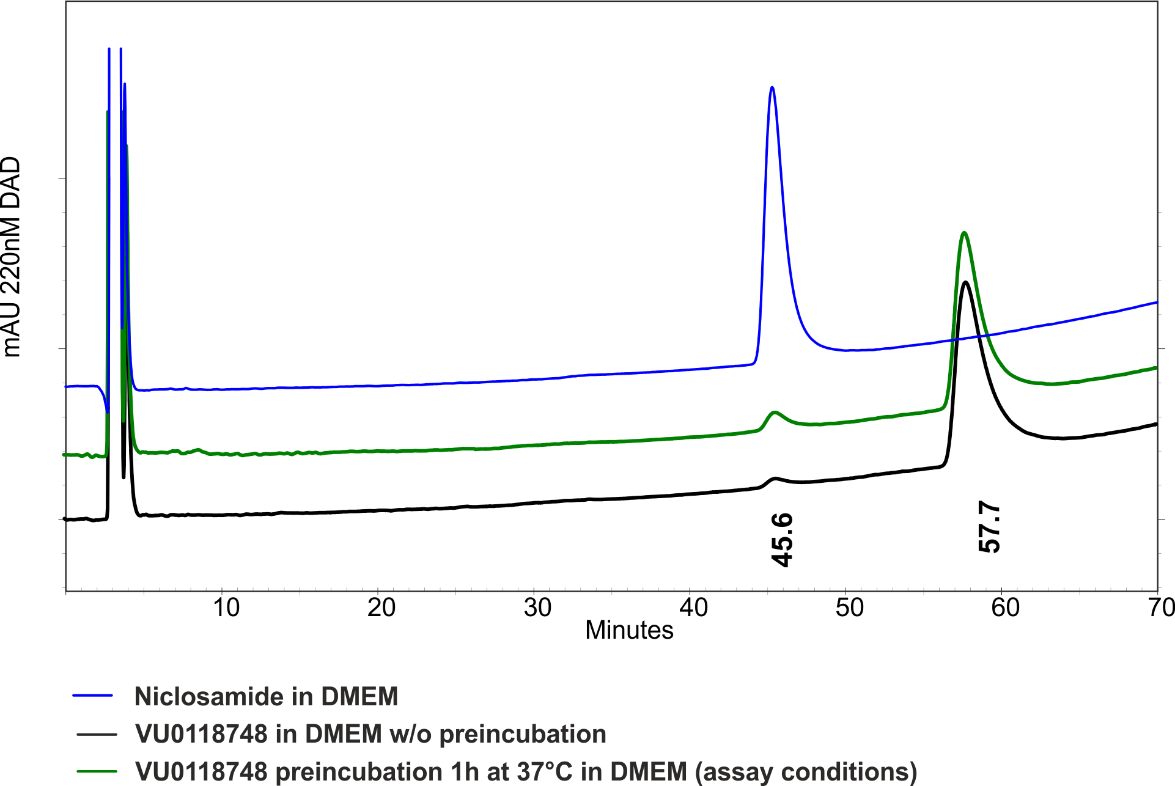

Supplement: S3 Fig — Niclosamide and VU0118748 showed different retention times of 46 min (63% ACN) and 58 min (71% ACN), respectively. VU0118748 in DMEM (without pre-incubation, black) shows a major signal with 98% integrated absorption at 58 min retention time, indicating the intact VU0118748. To test the stability of VU0118748 in the assay conditions, the compound was pre-incubated in DMEM for 2 hours at 37°C. HPLC analysis afterwards (green) shows a slightly increased fraction at 46 min retention time (5% of total absorption). Results show 95% of the compound is still intact. (PNG) [file pone.0157146.s003.png]
