# Supplementary material for: Discovery of Small-Molecule Modulators of the Human Y4 Receptor
Source: PLoS One. 2016 Jun 13;11(6):e0157146. doi: 10.1371/journal.pone.0157146 (PMC4905667; doi:10.1371/journal.pone.0157146)
Supplement: S1 Table — (PDF) [file pone.0157146.s004.pdf]

Supporting Information Table S1 Substructure search: Niclosamide analogues and results.

Backbone Modification	Search Structure	Tanimoto Cutoff	Total Compounds
None	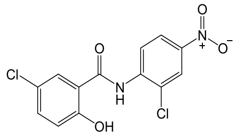	0.63	380
Urea	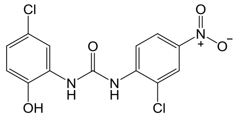	0.50	144
Thiourea	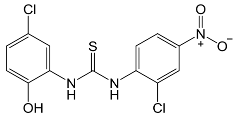	0.45	396
δ-lactam	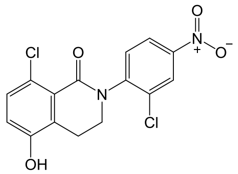	0.57	324
Methylene extension (a)	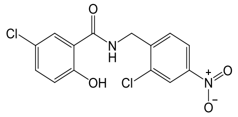	0.57	232
Methylene extension (b)	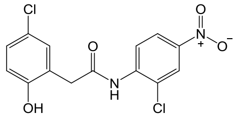	0.60	183
